# No Evidence of Association between Childhood Urban Environment and Cortical Thinning in Psychotic Disorder

**DOI:** 10.1371/journal.pone.0166651

**Published:** 2017-01-03

**Authors:** Aleida Frissen, Jim van Os, Ritsaert Lieverse, Petra Habets, Ed Gronenschild, Machteld Marcelis, Genetic Risk and Outcome in Psychosis (G.R.O.U.P.)

**Affiliations:** 1 Department of Psychiatry and Psychology, Maastricht University, Maastricht, The Netherlands; 2 King’s College London, King’s Health Partners, Department of Psychosis Studies, Institute of Psychiatry, London, United Kingdom; 3 Institute for Mental Health Care Eindhoven (GGzE), Eindhoven, The Netherlands; Kanazawa Daigaku, JAPAN

## Abstract

**Background:**

The alterations in cortical morphology, such as cortical thinning, observed in psychotic disorder, may be the outcome of interacting genetic and environmental effects. It has been suggested that urban upbringing may represent a proxy environmental effect impacting cortical thickness (CT). Therefore, the current study examined whether the association between group as a proxy genetic variable (patients with psychotic disorder [high genetic risk], healthy siblings of patients [intermediate risk] and healthy control subjects [average risk]) and CT was conditional on different levels of the childhood urban environment and whether this was sex-dependent.

**Methods:**

T1-weighted MRI scans were acquired from 89 patients with a psychotic disorder, 95 non-psychotic siblings of patients with psychotic disorder and 87 healthy control subjects. Freesurfer software was used to measure CT. Developmental urban exposure was classified as low, medium, and high, reflecting the population density and the number of moves between birth and the 15^th^ birthday, using data from the Dutch Central Bureau of Statistics and the equivalent database in Belgium. Multilevel regression analyses were used to examine the association between group, sex, and urban upbringing (as well as their interactions) and cortical CT as the dependent variable.

**Results:**

CT was significantly smaller in the patient group compared to the controls (B = -0.043, p <0.001), but not in the siblings compared to the controls (B = -0.013, p = 0.31). There was no main effect of developmental urbanicity on CT (B = 0.001, p = 0.91). Neither the three-way group × urbanicity × sex interaction (χ^2^ = 3.73, p = 0.16), nor the two-way group × urbanicity interaction was significant (χ^2^ = 0.51, p = 0.77).

**Conclusion:**

The negative association between (familial risk for) psychotic disorder and CT was not moderated by developmental urbanicity, suggesting that reduced CT is not the outcome of familial sensitivity to the proxy environmental factor ‘urban upbringing’.

## Introduction

Childhood urban environment is one of the (proxy) environmental risk factors for psychotic disorder. Both urban birth and urban upbringing have been found to contribute to the risk [1–5], with meta-analytic evidence for a dose-response relationship [3, 6, 7]. The vulnerable period of impact is specific to childhood and adolescence [8, 9] and the effect of the urban environment may be conditional on the genetic risk for psychotic disorder [10–12]. The urban environment is a proxy risk factor, likely reflecting a complex environment. The factors associated with the urban environment that mediate this effect, as well as the neurobiologic pathways involved, remain largely unknown. Candidate mechanisms include higher level of perceived social isolation in urban areas [13] and greater exposure to social "defeat" occasioned by higher level of competition in cities [14].

Reduced cortical thickness (CT) has frequently been observed in patients with psychotic disorder and to a certain extent also in their healthy relatives, suggesting that cortical thinning may represent neurodevelopmental alterations that result from a genetic liability for the disorder [15–21]. Regions frequently affected are the frontal, temporal and cingulate cortex [19, 21–25]. However, not all reports are consistent with regard to the presence of a CT intermediate phenotype. For example Boos and colleagues did not reveal cortical thinning in siblings of patients with schizophrenia [19, 26–28]. Thus, these studies suggest that the cortical abnormalities in psychotic disorder reflect a disease-related process or differential sensitivity to environmental risks. Indeed, there are some studies which support the hypothesis that reduced CT may be the outcome of differential sensitivity to the environment, though studies are scarce. For example, previous analyses on the current study sample showed that lower CT was associated with higher levels of cannabis use in both patients with psychotic disorder and siblings, and with higher levels of childhood trauma in patients, but not siblings [29]. Another study reported that fetal hypoxia was related to reduced gray matter volume and increased cerebrospinal fluid in patients with schizophrenia and their healthy relatives, but not in control [30]. To our knowledge, there are no studies with developmental urban exposure as a risk factor and structural brain measures as the outcome in patients with psychotic disorder. However, there are two studies examining the effect of developmental urbanicity in healthy volunteers: Haddad and colleagues showed that developmental urbanity was associated with a decrease in gray matter volume in the dorsolateral prefrontal cortex and a decrease in gray matter volume of the perigenual anterior cingulate cortex (pACC) in men only [31]; and Lederbogen and colleagues found that urban upbringing was related to altered activity of the perigenual anterior cingulate cortex, a key region for the regulation of negative affect and stress [32].

Developmental urbanicity has been linked to early life stress through higher levels of social isolation [13] and social defeat [14]. Such a form of early life stress may be associated with cerebral alterations, as suggested by evidence from human [33–35] and animal studies [36–39]. The underlying biological mechanisms may comprise hypothalamic-pituitary-adrenal axis (HPA-axis) alterations, which could have direct effects (through increased cortisol levels [40]) and indirect effects (by affecting dopaminergic circuits [41, 42]). Excessive dopamine (DA) is associated with DNA damage and apoptosis [43, 44] and stimulation of the D1 dopamine receptor might also cause neurodegeneration the striatum [45].

In conclusion, CT alterations may be the consequence of underlying operational mechanisms, such as gene-environment interactions. The hypothesis of this study was that the association between CT, the outcome of interest in the current study, and group (patients with psychotic disorder, healthy siblings of patients and healthy control subjects), is conditional on exposure to developmental urbanicity in patients with psychotic disorder (highest genetic risk) and, to a lesser degree, in their healthy siblings (intermediate genetic risk). This phenomenon would thus be suggestive of gene-environment interaction (GXE), or genetic control of sensitivity to the environment. Given that structural brain alterations may be more prominent in male patients [46], interactions with sex were included in the analyses.

## Materials and Methods

### Participants

Data pertain to baseline measures of a longitudinal magnetic resonance imaging (MRI) study in Maastricht, the Netherlands. The sample consisted of 89 patients with a diagnosis of a non-affective psychotic disorder, 95 siblings, and 87 control subjects. Inclusion criteria for the patient group were the following: (1) age 16 to 50 years; (2) diagnosis of non-affective psychotic disorder; and (3) sufficient command of the Dutch language. Sibling non-patient status was defined as the absence of any lifetime psychotic disorder. Controls had no first-degree relative with a psychotic disorder as established by the Family Interview for Genetic Studies [47] with the control as informant. The sample included 63 families, of which 39 families contributed one patient and one discordant sibling, two families contributed one patient and two discordant siblings, and two families contributed one patient and three discordant siblings. Two families contributed two patients, seven families contributed two discordant siblings, and one family contributed three discordant siblings. In the control group, ten families contributed two siblings. In addition, 42 independent patients, 29 independent siblings, and 66 independent control subjects were included.

Diagnosis was based on DSM-IV criteria [48], assessed with the Comprehensive Assessment of Symptoms and History (CASH) interview [49]. The CASH was also used to confirm the absence of a diagnosis of nonaffective psychosis in the siblings and absence of a lifetime diagnosis of any psychotic disorder or any current affective disorder in the healthy control subjects.

Before MRI acquisition, participants were screened for the following exclusion criteria: (1) brain injury (unconsciousness > 1 hour); (2) meningitis or other neurological diseases that might have affected brain structure or function; (3) cardiac arrhythmia requiring medical treatment; and (4) severe claustrophobia. In addition, subjects with metal corpora aliena were excluded from the study, as were women with intrauterine device status and (suspected) pregnancy. The selection procedure and the in- and exclusion criteria are described more elaborate in a previous paper [29].

The study was approved centrally by the Ethical Review Board of the University Medical Centre Utrecht. Written informed consent was obtained from all subjects after they (1) read a document with detailed information about the nature and possible consequences of the study; (2) had verbally discussed any possible concerns with the researcher; and (3) had provided clear indication that they had understood the procedure. In the Netherlands, adult patients with mental illness are considered participating citizens who have the right to make independent informed decisions including the autonomous decision to participate in research; therefore consent of relatives was not sought(as described previously [29]).

### Measures

The level of symptoms in all groups was assessed with the Positive and Negative Syndrome Scale (PANSS) [50]. Educational level was defined as highest accomplished level of education. In the patient group, antipsychotic (AP) medication use was determined by the patient's report and verified with the treating consultant psychiatrist. Best estimate lifetime (cumulative) AP use was determined by multiplying the number of days of AP use with the corresponding haloperidol equivalents and summing these scores for all periods of AP use (including the exposure period between baseline assessment for the G.R.O.U.P. study and the moment of baseline MRI scanning), using the recently published converting formulas for AP dose equivalents described by Andreasen et al [51].

Substance use was measured with the Composite International Diagnostic Interview (CIDI) sections B—J—L [52]. Use of cannabis and other drugs was assessed as reported frequency of use during the last 12 months, as well as lifetime use. CIDI frequency data on cannabis and other drug use was available for respectively 263 participants (3% missing data) and 267 participants (1.5% missing data). Alcohol use was defined as the reported number of weekly consumptions during the last 12 months. CIDI frequency data on alcohol use were available 235 participants (13.3% missing data).

Childhood trauma was assessed with the Dutch version of the Childhood Trauma Questionnaire Short Form (CTQ) [53]. The short CTQ consists of 25 items rated on a 5-point Likert scale (1 = never true to 5 = very often true) inquiring about traumatic experiences in childhood. Five types of childhood maltreatment were assessed: emotional, physical and sexual abuse, and emotional and physical neglect, with five questions covering each type of trauma. As a general measure of childhood trauma a summary score was created by adding the mean scores of each trauma type. The CTQ data were missing for two persons (0.7% missing data), as described in a previous study [29].

### Level of urbanicity

We generated a historical population density record for each municipality from 1930 onwards using the Dutch Central Bureau of Statistics (CBS) and equivalent Belgium database. When data was not available, linear extrapolations were computed. When historical names of municipalities disappeared from historical records (e.g. due to city mergers) we used the available data from the agglomerate city. Subjects were asked to describe where they lived during their life. This resulted in a number of records for each subject, containing locations by age period. For each of these records, we computed the average population density (by square kilometer, excluding water) of the municipality for the matching year periods. Subsequently, it was determined where the subject lived at birth, between ages 0–4 years; between 5–9 years; 10–14 years; 15–19 years; 20–39 years; 40–59 years; and 60+ up to the actual age. Average population density over the period was categorized conform the Dutch CBS urbanicity rating (1 = <500/km^2^; 2 = 500–1000/km^2^; 3 = 1000–1500/km^2^; 4 = 1500–2500/km^2^; 5 = 2500+/km^2^). The periods 0–4 years, 5–9 years and 10–14 years were merged to average urbanicity exposure between 0–14 years. The latter divided by its tertiles was used as the primary variable reflecting developmental urbanicity exposure (low, medium, high) in the analyses. Urbanicity data were available for 263 participants (3.0% missing data). This urbanicity measures was also described in a previous paper [54].

### MRI acquisition and processing

The MRI scans were acquired using a 3T Siemens scanner (Erlangen, Germany) and the following acquisition parameters: Modified Driven Equilibrium Fourier Transform (MDEFT) sequence = 176 slices, 1 mm isotropic voxel size, echo time 2.4 ms, repetition time 7.92 ms, inversion time 910 ms, flip angle 15°, total acquisition time 12 min 51 s; Magnetization Prepared Rapid Acquisition Gradient-Echo (MPRAGE; Alzheimer’s Disease Neuroimaging Initiative) sequence = 192 slices, 1 mm isotropic voxel size, echo time 2.6 ms, repetition time 2250 ms, inversion time 900 ms, flip angle 9°, total acquisition time 7 min 23 s. For both anatomical scans, the matrix size was 256 × 256 and field of view was 256 × 256 mm^2^. The number of excitations was one. Two sequences were used because of a scanner update during data collection. The total proportion of MPRAGE scans did not differ between the groups (χ^2^ = 4.95, p = 0.08).

### Cortical thickness measurement

Scans were processed and analyzed using Freesurfer stable release v5.3.0, http://surfer.nmr.mgh.harvard.edu [55–61]. To measure CT, the cerebral cortex was parcellated into units based on gyral and sulcal structure [62, 63]. Furthermore, a variety of surface-based data was created including maps of curvature and sulcal depth. This method used both intensity and continuity information from the entire three dimensional MR volume in segmentation and deformation procedures to produce representations of CT, calculated as the closest distance from the grey/white matter boundary to the grey matter/CSF boundary at each vertex on the tessellated surface [56]. The maps were created using spatial intensity gradients across tissue classes and were not restricted to the voxel resolution of the original data, thus were capable of detecting sub-millimeter differences between groups. CT measurement procedures have been validated against histological analysis [64] and manual measurements [18, 65]. Per hemisphere there are 34 regions (68 in total) of interest (adapted from the Desikant atlas) [62]; for each of these regions, CT measurements were derived by FreeSurfer and exported to Stata and R version 3.2.1. Thus, every individual had 68 CT measurements over the predefined ROIs. These procedures are described in detail in a previous paper [29].

### Statistical/data analyses

The CT data consist of multiple observations nested within persons and within families, generating 3 levels of analysis: 68 regional CT measures (level 1) nested in subjects (level 2) who were part of the same families (level 3) [66]. Given the multilevel hierarchical structure of the data, compromising statistical independence of the observations, multilevel linear regression analyses were performed with CT as the dependent variable and subject number and family ID as random effects in all analyses. R version 3.2.1 [67] was used, employing the nlme, car and multcomp packages. Since the outcome (CT) represents means (based on varying numbers of vertices), the analyses were weighted based on the number of vertices per region. The regression coefficients (B) from the multilevel linear regression model represent the effect size of the predictors and can be interpreted identically to estimates in equivalent unilevel linear regression analyses. In order to check assumption of normality qq-plots of the residuals were inspected, which showed an even distribution. Also, the spread of standardized residuals against fitted values was evaluated, which was constant, i.e. there was no evidence of heteroscedasticity.

In order to study whether there is differential sensitivity to developmental urbanicity in the three groups and in both sexes, a three-way interaction between group (healthy control subject = 0, sibling = 1 or patient = 2, entered as linear variable and as dummy variables with healthy control subject as the reference category), sex (male = 0 or female = 1) and developmental urbanicity (low = 0, medium = 1 or high = 2, entered both as a linear variable and as dummy variables with ‘low’ urbanicity as the reference category) was added to the model. Main effects and interaction terms were tested using the chi-square test. The statistical model was: CT = β_0_ + β_1_(group) + β_2_(sex) + β_3_(urbanicity) + β_4_ (group × sex) + β_5_ (group × urbanicity) + β_6_ (sex × urbanicity) + β_7_ (group × sex × urbanicity). First, the three-way interaction was tested, and subsequently all the two-way interactions. Group was modelled both as linear and as dummy variables, with healthy control subjects as the reference category, in order to examine the level of dose-response. Developmental urbanicity was similarly entered both as a linear variable and as dummy variables, with ‘low’ urbanicity as the reference category, in order to examine the level of dose-response. In the case of significant interaction, individual slopes were calculated by combination of effects from the model and tested whether they differed significantly. All analyses were adjusted for the a priori selected confounders: age, sex, highest accomplished level of education and scantype (MDEFT or MPRAGE). The model was also analyzed with cannabis and childhood trauma as additional confounders, because cannabis and childhood trauma have been related to changes in CT in psychotic disorder [29] and to risk for psychotic disorder.

## Results

### Participant characteristics

All relevant data are provided in a supporting information file. Sociodemographic and clinical characteristics of the sample are summarized in Table 1. The patient group comprised more men and level of education was lowest in the patient group compared to controls and siblings. Seventy-four out of 89 patients were currently using AP medication (first generation AP: n = 3; second generation AP: n = 71), 16 patients used antidepressants, nine used benzodiazepines, six used antiepileptic medication and three used lithium. Two control subjects and three siblings used antidepressants, and one sibling used benzodiazepines. Cumulative lifetime AP exposure was not significantly associated with CT in the patient group (B < 0.001, p = 0.55).

**Table 1:** Participant demographics.

	Patients (n = 89)	Sibling (n = 95)	Controls (n = 87)
Age at scan	28.1 ± 7.0	29.5 ± 8.8	30.8 ± 10.8
Sex n (%), male	60 (67%)	49 (52%)	33 (38%)
Level of education	4.3 ± 2.0	5.1 ± 2.0	5.4 ± 1.8
Cannabis lifetime^a^	45.0 ± 46.7	20.0 ± 37.7	7.4 ± 21.1
Other drug lifetime^a^	44.6 ± 88.7	6.1 ± 31.3	2.0 ± 11.6
Alcohol use units/week^b^	4.9 ± 9.0	9.6 ± 17.1	5.0 ± 7.0
Childhood urbanicity^c^	2.4 ± 1.3	2.3 ± 1.4	2.4 ± 1.5
Childhood trauma^d^	7.2 ± 2.8	5.8 ± 1.5	5.7 ± 1.8
PANSS Positive	10.5 ± 5.0	7.4 ± 1.5	7.3 ± 1.1
PANSS Negative	12.0 ± 5.7	8.5 ± 2.1	8.2 ± 1.0
PANSS Disorganization	12.5 ± 4.0	10.4 ± 0.9	10.2 ± 1.1
PANSS Excitement	9.8 ± 2.7	8.6 ± 1.4	8.3 ± 1.1
PANSS Emotional Distress	13.2 ± 5.2	10.0 ± 2.8	9.2 ± 2.0
Age of onset	21.7 ± 7.0	-	-
Antipsychotics^e^	6777.4 ± 6152.5	-	-

PANSS = Positive and negative syndrome scale.

^a^ Mean number of times

^b^ Weekly consumptions on the last 12 months.

^c^ Mean level of childhood urban exposure, Five levels of urbanicity/population density 1 = <500 inhabitants/km^2^; 2 = 500–1000 inhabitants/km^2^; 3 = inhabitants 1000–1500/km^2^; 4 = inhabitants 1500–2500/km^2^; 5 = 2500+/km^2^.

^d^ Summary score of Childhood Trauma Questionnaire Short Form (CTQ)

^e^ Lifetime exposure in haloperidol equivalents

### Three-way interaction with group, sex and developmental urbanicity in the model of CT

The three-way interaction between group (linear variable and dummy variables), urbanicity (linear variable and dummy variables) and sex, in the model of CT was not significant (group linear variable × urbanicity linear variable × sex: χ^2^ = 3.14, p = 0.08; group linear variable × urbanicity dummy variables × sex: χ^2^ = 2.88, p = 0.24; group dummy variables × urbanicity linear variable × sex: χ^2^ = 3.73, p = 0.16; group dummy variables × urbanicity dummy variables x sex: χ^2^ = 3.08, p = 0.55), indicating that the association between CT and group was not moderated by developmental urbanicity and sex (see Table 2). Results did not change significantly when cannabis and childhood trauma were included in the analysis.

**Table 2:** Cortical Thickness as a Function of Group Status, Sex and Urbanicity.

	Urbanicity level	Patients		Relatives		Controls	
		No. obs (n)^a^	CT^b^	No. obs (n)	CT	No. obs (n)	CT
Male	Low	1360 (20)	2.50 ± 0.39	1224 (18)	2.47 ± 0.40	680 (10)	2.51 ± 0.42
	Medium	1564 (23)	2.50 ± 0.41	884 (13)	2.51 ± 0.38	816 (12)	2.56 ± 0.39
	High	1088 (16)	2.49 ± 0.41	1156 (17)	2.48 ± 0.38	612 (9)	2.49 ± 0.40
female	Low	340 (5)	2.47 ± 0.39	1156 (17)	2.53 ± 0.40	1564 (23)	2.50 ± 0.40
	Medium	680 (10)	2.52 ± 0.41	1156 (17)	2.54 ± 0.39	680 (10)	2.52 ± 0.39
	High	884 (13)	2.50 ± 0.41	680 (10)	2.55 ± 0.42	1360 (20)	2.48 ± 0.38

^a^ No. obs = number of subjects × number of regions (68); numbers in parentheses indicate number of subjects.

^b^ Mean ± SD, Means are raw means not adjusted for covariates.

### Two-way interaction between group and developmental urbanicity in the model of CT

There was no significant group (linear variable and dummy variables) × urbanicity (linear variable and dummy variables) interaction (group linear variable × urbanicity linear variable: χ^2^ = 0.43, p = 0.51; group linear variable × urbanicity dummy variables: χ^2^ = 2.29, p = 0.32; group dummy variables × urbanicity linear variable: χ^2^ = 0.51, p = 0.77; group dummy variables × urbanicity dummy variables: χ^2^ = 4.23, p = 0.38). Table 3 shows the mean values of CT per group and urbanicity level. There was no consistent pattern of reduction or increase in CT with increasing levels of developmental urbanicity in any group. Adding cannabis and childhood trauma as confounders did not change the results.

**Table 3:** Cortical Thickness as a Function of Group Status and Urbanicity.

	Patients		Relatives		Controls	
Urbanicity level	No. obs (n)^a^	CT^b^	No. obs (n)	CT	No. obs (n)	CT
Low	1700 (25)	2.50 ± 0.39	2380 (35)	2.50 ± 0.40	2244 (33)	2.51 ± 0.40
Medium	2244 (33)	2.51 ± 0.41	2040 (30)	2.53 ± 0.39	1496 (22)	2.54 ± 0.39
High	1972 (29)	2.50 ± 0.41	1836 (27)	2.50 ± 0.40	1972 (29)	2.49 ± 0.38

^a^ No. obs = number of subjects × number of regions (68); numbers in parentheses indicate number of subjects.

^b^ Mean ± SD, Means are raw means not adjusted for covariates.

The other two-way interactions, group × sex and urbanicity × sex, were also not significant (group × sex [both dummy variables]: χ^2^ = 0.73, p = 0.69; urbanicity [linear variable] × sex (dummy variables): χ^2^ = 0.35, p = 0.55).

### Main effects of group and urbanicity on CT

CT was significantly smaller in the patients compared to the controls and siblings (P vs. Con: B = -0.043, p<0.001; P vs. Sib: B = -0.031, p = 0.006). The siblings were not significantly different from the controls (B = -0.013, p = 0.31). There was neither a significant association between sex and CT (B = 0.004, p = 0.71), nor between developmental urbanicity (linear variable) and CT (B < 0.001, p = 0.91). See Table 4.

**Table 4:** Cortical Thickness by Group and Urbanicity level.

	No. of observations^a^	Cortical thickness Mean^b^ ± SD	B^c^	p
**Group**				
Controls	5916 (87)	2.51 ± 0.40		
Siblings	6460 (95)	2.51 ± 0.40	-0.013	0.31
Patients	6052 (89)	2.50 ± 0.40	-0.043	<0.01
B linear trend^d^			-0.022	<0.01
**Urbanicity**				
Low	6324 (93)	2.50 ± 0.40		
Medium	5780 (85)	2.52 ± 0.40	0.001	0.96
High	5780 (85)	2.50 ± 0.40	0.001	0.91
B linear trend^d^			0.001	0.91

^a^ No. of observations = number of subjects × number of regions (68); numbers in parentheses indicate number of subjects.

^b^ Means are raw means not adjusted for covariates.

^c^ B represent the regression coefficients from multilevel linear regression analyses, adjusted for age, sex, cannabis use and highest level of education, for group controls were the reference category and for urbanicity ‘low’ urbanicity level was the reference category

^d^ B linear trend represents the summary change in CT with one unit increase in exposure level (group and urbanicity)

### Post-hoc subgroup analysis

Subgroup analysis in which only 50% of the patients with the highest PANSS positive scores were included (n = 44), did not affect the pattern of the findings. The three-way interaction between urbanicity (linear variable and dummy variables), group and sex, and the two-way interaction between urbanicity (linear variable and dummy variables) and group were not significant (linear variable: three-way interaction: χ^2^ = 4.97, p = 0.08; two-way interaction: χ^2^ = 1.38, p = 0.50; dummy variables: three-way interaction: χ^2^ = 5.20, p = 0.27; two-way interaction: χ^2^ = 5.51, p = 0.24).

## Discussion

The current study showed that urban upbringing was not associated with reductions of CT in individuals with (risk for) psychotic disorder or healthy controls.

To our knowledge no previous studies on the association between developmental urban exposure levels and CT as a function of different levels of psychosis risk are available. However, an fMRI study linked early life urbanicity exposure to differential activity of the pACC in social stress processing in healthy students [32], suggesting that social stress mediates the impact of urbanicity on neural circuits, although other mechanisms can be envisaged. In the current study, there was no evidence for CT alterations in the pACC and the cingulate cortex. However, absence of structural alterations does not automatically imply absence of functional alterations: cell composition, cell activity or connectivity may be different despite normal volumes of discrete brain areas.

Epidemiological studies demonstrate that there is reason to believe that the relationship between urban upbringing and psychotic disorders is causal: (1) the findings are consistent across countries and cultures; (2) there is evidence of a dose-response relationship; and (3) urban upbringing precedes psychotic disorder [3]. The current study revealed that CT reductions in patients with psychotic disorder are not moderated by developmental urbanicity. An explanation for the absence of an association between cortical thinning and developmental urbanicity in psychotic disorder is that urban upbringing may influence different neurobiologic pathways that do not imply CT alteration. For example, changes in white matter could be involved or the nature of change could be functional instead of structural. In case of the latter, a potential pathologic effect of the proxy environmental risk factor associated with urbanicity on brain structure may be identified with functional MRI [32].

The absence of an association could also be explained by a small urban-rural difference. Our participants grew up in the Netherlands, which can be described as a relative safe and well developed country compared to certain other counties where the urban-rural discrepancies may be more prominent. However, studies conducted in the Netherlands did report a higher incidence of psychotic disorders related to urban upbringing [8, 68], indicating that although the difference is relatively small, it is still large enough to show an effect. Another explanation of the absence of a significant association could be that the number of participants in the higher urbanicity levels was relatively small compared to the lower urbanicity levels, resulting in reduced power.

Environmental factors for psychotic disorder, other than the urban exposure, have been related to grey matter reductions. For instance, a study by Cannon et al. [30] found that a history of fetal hypoxia is associated with gray matter volume reductions among patients with schizophrenia and their healthy siblings, but not among healthy controls. A plausible explanation for the relationship between this environmental exposure and gray matter reductions is that fetal hypoxia is more likely to have a direct effect on neurodevelopment than the developmental urban exposure since more than 50% of the candidate genes for susceptibility to schizophrenia are subject to regulation by hypoxia [69]. In addition, fetal hypoxia has been associated with increased brain dopamine synthesis capacity and concentrations [70]. In previous analyses on (partly) the same study sample as the current one, Habets and colleagues [29] found that higher levels of cannabis consumption (lifetime) and childhood trauma experiences were associated with increased cortical thinning in patients with psychotic disorder, but not in controls. In siblings, the same pattern was found for cannabis, but not for childhood trauma. Just as fetal hypoxia, cannabis exposure and developmental trauma have been more directly related to altered neurodevelopment than developmental urbanicity. Tetrahydrocannabinol (THC), the active psychotropic ingredient of cannabis, may lead to increased striatal dopamine levels [71]. THC is also believed to affect synaptic plasticity and it may affect gray matter volume through a mechanism on neurotoxicity [71, 72]. Childhood trauma is thought to affect dopaminergic circuits through altered functioning of the hypothalamic-pituitary-adrenal (HPA) axis [41, 42]. In conclusion, the developmental urbanicity exposure may be too crude compared to the other environmental risk factors to increase cerebral cortical vulnerability in individuals with (risk for) psychotic disorder.

### Methodological considerations

This is the first study examining CT as the outcome of developmental urbanicity exposure and psychosis risk, in a relatively large study sample. Our study population is larger than most previous studies on CT in psychotic disorder [16, 18, 20, 22], although some studies had larger sample sizes e.g. the study by Goldman and colleagues [19]. Further, the absence of a main effect of urbanicity does not necessarily imply insufficient power for interaction: environmental risks may only become apparent when examined in a G × E model.

Population density was used as a measure of developmental urbanicity, but it is not clear whether this is a good measure of urban exposure. When the factors mediating the effect of the urbanicity exposure could be identified, the chance of finding underlying neuropathologic mechanisms may increase. Future research is needed to unravel the factors related to the urban environment that mediate the higher risk for psychotic disorders.

Systematic reviews and a longitudinal study [51, 73–75] suggest that AP treatment contributes to the brain structural changes observed in psychotic disorders. However, there was no evidence for an association between cumulative AP dose and CT in the current sample. It is thus unlikely that this has influenced our results.

## Supporting Information

S1 Data Supporting Data. (XLS)
