# Correction: No Evidence of Association between Childhood Urban Environment and Cortical Thinning in Psychotic Disorder

**DOI:** 10.1371/journal.pone.0178312

**Published:** 2017-05-18

**Authors:** The PLOS ONE Staff

## Notice of Republication

This article was republished on February 14, 2017, to correct an error in the author list. The publisher apologizes for the error. Please download the article again to view the correct version. The originally published, uncorrected article and the republished, corrected articles are provided here for reference.

## Supporting information

S1 File Originally published, uncorrected article. (PDF)

S2 File Republished, corrected article. (PDF)
