# Supplementary material for: Correction: No Evidence of Association between Childhood Urban Environment and Cortical Thinning in Psychotic Disorder
Source: PLoS One. 2017 May 18;12(5):e0178312. doi: 10.1371/journal.pone.0178312 (PMC5436876; doi:10.1371/journal.pone.0178312)
Supplement: S2 File — (PDF) [file pone.0178312.s002.pdf]

PLOS ONE

RESEARCH ARTICLE

No Evidence of Association between Childhood Urban Environment and Cortical Thinning in Psychotic Disorder

Aleida Frissen[1], Jim van Os[1,2], Ritsaert Lieverse[1], Petra Habets[1], Ed Gronenschild[1], Machteld Marcelis[1,3]*, Genetic Risk and Outcome in Psychosis (G.R.O.U.P.)[¶]

1 Department of Psychiatry and Psychology, Maastricht University, Maastricht, The Netherlands, **2** King's College London, King's Health Partners, Department of Psychosis Studies, Institute of Psychiatry, London, United Kingdom, **3** Institute for Mental Health Care Eindhoven (GGzE), Eindhoven, The Netherlands

¶ Membership of G.R.O.U.P is provided in the Acknowledgments.
* m.marcelis@maastrichtuniversity.nl

OPEN ACCESS

Citation: Frissen A, van Os J, Lieverse R, Habets P, Gronenschild E, Marcelis M, et al. (2017) No Evidence of Association between Childhood Urban Environment and Cortical Thinning in Psychotic Disorder. PLoS ONE 12(1): e0166651. doi:10.1371/journal.pone.0166651

Editor: Takanori Hashimoto, Kanazawa Daigaku, JAPAN

Received: May 12, 2016

Accepted: November 1, 2016

Published: January 3, 2017

Copyright: © 2017 Frissen et al. This is an open access article distributed under the terms of the Creative Commons Attribution License, which permits unrestricted use, distribution, and reproduction in any medium, provided the original author and source are credited.

Data Availability Statement: Data pertain to baseline measures of a longitudinal magnetic resonance imaging (MRI) study in Maastricht, the Netherlands, which is part of the the Genetic Risk and Outcome in Psychosis (G.R.O.U.P.) project. Authors may be contacted at Maastricht University Medical Centre, PO BOX 616 (location VIJV1), 6200 MD Maastricht The Netherlands, T 31-43-3688664.

Funding: This work was sponsored by the Dutch organization for scientific research NWO (Genetic

Abstract

Background

The alterations in cortical morphology, such as cortical thinning, observed in psychotic disorder, may be the outcome of interacting genetic and environmental effects. It has been suggested that urban upbringing may represent a proxy environmental effect impacting cortical thickness (CT). Therefore, the current study examined whether the association between group as a proxy genetic variable (patients with psychotic disorder [high genetic risk], healthy siblings of patients [intermediate risk] and healthy control subjects [average risk]) and CT was conditional on different levels of the childhood urban environment and whether this was sex-dependent.

Methods

T1-weighted MRI scans were acquired from 89 patients with a psychotic disorder, 95 non-psychotic siblings of patients with psychotic disorder and 87 healthy control subjects. Free-surfer software was used to measure CT. Developmental urban exposure was classified as low, medium, and high, reflecting the population density and the number of moves between birth and the 15th birthday, using data from the Dutch Central Bureau of Statistics and the equivalent database in Belgium. Multilevel regression analyses were used to examine the association between group, sex, and urban upbringing (as well as their interactions) and cortical CT as the dependent variable.

Results

CT was significantly smaller in the patient group compared to the controls ($B = -0.043$, $p < 0.001$), but not in the siblings compared to the controls ($B = -0.013$, $p = 0.31$). There was no main effect of developmental urbanicity on CT ($B = 0.001$, $p = 0.91$). Neither the three-way group × urbanicity × sex interaction ($\chi^2 = 3.73$, $p = 0.16$), nor the two-way group × urbanicity interaction was significant ($\chi^2 = 0.51$, $p = 0.77$).

Risk and Outcome of Psychosis [G.R.O.U.P.]) and the European Community's Seventh Framework Programme under Grant Agreement No. HEALTH-F2-2009-241909 (European Network of National Schizophrenia Networks Studying Gene-Environment Interactions Consortium). The funders had no role in study design, data collection and analysis, decision to publish, or preparation of the manuscript.

Competing Interests: Jim van Os is or has been, in the last three years, an unrestricted research grant holder with, or has received financial compensation as an independent symposium speaker from Lundbeck and Janssen. Machteld Marcelis has received, in the last three years, financial compensation as an independent symposium speaker from Lundbeck and Janssen. All other authors report no biomedical financial interests or potential conflicts of interest. This does not alter our adherence to PLOS ONE policies on sharing data and materials.

Conclusion

The negative association between (familial risk for) psychotic disorder and CT was not moderated by developmental urbanicity, suggesting that reduced CT is not the outcome of familial sensitivity to the proxy environmental factor 'urban upbringing'.

Introduction

Childhood urban environment is one of the (proxy) environmental risk factors for psychotic disorder. Both urban birth and urban upbringing have been found to contribute to the risk [1–5], with meta-analytic evidence for a dose-response relationship [3, 6, 7]. The vulnerable period of impact is specific to childhood and adolescence [8, 9] and the effect of the urban environment may be conditional on the genetic risk for psychotic disorder [10–12]. The urban environment is a proxy risk factor, likely reflecting a complex environment. The factors associated with the urban environment that mediate this effect, as well as the neurobiologic pathways involved, remain largely unknown. Candidate mechanisms include higher level of perceived social isolation in urban areas [13] and greater exposure to social "defeat" occasioned by higher level of competition in cities [14].

Reduced cortical thickness (CT) has frequently been observed in patients with psychotic disorder and to a certain extent also in their healthy relatives, suggesting that cortical thinning may represent neurodevelopmental alterations that result from a genetic liability for the disorder [15–21]. Regions frequently affected are the frontal, temporal and cingulate cortex [19, 21–25]. However, not all reports are consistent with regard to the presence of a CT intermediate phenotype. For example Boos and colleagues did not reveal cortical thinning in siblings of patients with schizophrenia [19, 26–28]. Thus, these studies suggest that the cortical abnormalities in psychotic disorder reflect a disease-related process or differential sensitivity to environmental risks. Indeed, there are some studies which support the hypothesis that reduced CT may be the outcome of differential sensitivity to the environment, though studies are scarce. For example, previous analyses on the current study sample showed that lower CT was associated with higher levels of cannabis use in both patients with psychotic disorder and siblings, and with higher levels of childhood trauma in patients, but not siblings [29]. Another study reported that fetal hypoxia was related to reduced gray matter volume and increased cerebrospinal fluid in patients with schizophrenia and their healthy relatives, but not in control [30]. To our knowledge, there are no studies with developmental urban exposure as a risk factor and structural brain measures as the outcome in patients with psychotic disorder. However, there are two studies examining the effect of developmental urbanicity in healthy volunteers: Haddad and colleagues showed that developmental urbanity was associated with a decrease in gray matter volume in the dorsolateral prefrontal cortex and a decrease in gray matter volume of the perigenual anterior cingulate cortex (pACC) in men only [31]; and Lederbogen and colleagues found that urban upbringing was related to altered activity of the perigenual anterior cingulate cortex, a key region for the regulation of negative affect and stress [32].

Developmental urbanicity has been linked to early life stress through higher levels of social isolation [13] and social defeat [14]. Such a form of early life stress may be associated with cerebral alterations, as suggested by evidence from human [33–35] and animal studies [36–39]. The underlying biological mechanisms may comprise hypothalamic-pituitary-adrenal axis (HPA-axis) alterations, which could have direct effects (through increased cortisol levels [40]) and indirect effects (by affecting dopaminergic circuits [41, 42]). Excessive dopamine (DA) is

associated with DNA damage and apoptosis [43, 44] and stimulation of the D1 dopamine receptor might also cause neurodegeneration the striatum [45].

In conclusion, CT alterations may be the consequence of underlying operational mechanisms, such as gene-environment interactions. The hypothesis of this study was that the association between CT, the outcome of interest in the current study, and group (patients with psychotic disorder, healthy siblings of patients and healthy control subjects), is conditional on exposure to developmental urbanicity in patients with psychotic disorder (highest genetic risk) and, to a lesser degree, in their healthy siblings (intermediate genetic risk). This phenomenon would thus be suggestive of gene-environment interaction (GXE), or genetic control of sensitivity to the environment. Given that structural brain alterations may be more prominent in male patients [46], interactions with sex were included in the analyses.

Materials and Methods

Participants

Data pertain to baseline measures of a longitudinal magnetic resonance imaging (MRI) study in Maastricht, the Netherlands. The sample consisted of 89 patients with a diagnosis of a non-affective psychotic disorder, 95 siblings, and 87 control subjects. Inclusion criteria for the patient group were the following: (1) age 16 to 50 years; (2) diagnosis of non-affective psychotic disorder; and (3) sufficient command of the Dutch language. Sibling non-patient status was defined as the absence of any lifetime psychotic disorder. Controls had no first-degree relative with a psychotic disorder as established by the Family Interview for Genetic Studies [47] with the control as informant. The sample included 63 families, of which 39 families contributed one patient and one discordant sibling, two families contributed one patient and two discordant siblings, and two families contributed one patient and three discordant siblings. Two families contributed two patients, seven families contributed two discordant siblings, and one family contributed three discordant siblings. In the control group, ten families contributed two siblings. In addition, 42 independent patients, 29 independent siblings, and 66 independent control subjects were included.

Diagnosis was based on DSM-IV criteria [48], assessed with the Comprehensive Assessment of Symptoms and History (CASH) interview [49]. The CASH was also used to confirm the absence of a diagnosis of nonaffective psychosis in the siblings and absence of a lifetime diagnosis of any psychotic disorder or any current affective disorder in the healthy control subjects.

Before MRI acquisition, participants were screened for the following exclusion criteria: (1) brain injury (unconsciousness > 1 hour); (2) meningitis or other neurological diseases that might have affected brain structure or function; (3) cardiac arrhythmia requiring medical treatment; and (4) severe claustrophobia. In addition, subjects with metal corpora aliena were excluded from the study, as were women with intrauterine device status and (suspected) pregnancy. The selection procedure and the in- and exclusion criteria are described more elaborate in a previous paper [29].

The study was approved centrally by the Ethical Review Board of the University Medical Centre Utrecht. Written informed consent was obtained from all subjects after they (1) read a document with detailed information about the nature and possible consequences of the study; (2) had verbally discussed any possible concerns with the researcher; and (3) had provided clear indication that they had understood the procedure. In the Netherlands, adult patients with mental illness are considered participating citizens who have the right to make independent informed decisions including the autonomous decision to participate in research; therefore consent of relatives was not sought(as described previously [29]).

Measures

The level of symptoms in all groups was assessed with the Positive and Negative Syndrome Scale (PANSS) [50]. Educational level was defined as highest accomplished level of education. In the patient group, antipsychotic (AP) medication use was determined by the patient's report and verified with the treating consultant psychiatrist. Best estimate lifetime (cumulative) AP use was determined by multiplying the number of days of AP use with the corresponding haloperidol equivalents and summing these scores for all periods of AP use (including the exposure period between baseline assessment for the G.R.O.U.P. study and the moment of baseline MRI scanning), using the recently published converting formulas for AP dose equivalents described by Andreasen et al [51].

Substance use was measured with the Composite International Diagnostic Interview (CIDI) sections B—J—L [52]. Use of cannabis and other drugs was assessed as reported frequency of use during the last 12 months, as well as lifetime use. CIDI frequency data on cannabis and other drug use was available for respectively 263 participants (3% missing data) and 267 participants (1.5% missing data). Alcohol use was defined as the reported number of weekly consumptions during the last 12 months. CIDI frequency data on alcohol use were available 235 participants (13.3% missing data).

Childhood trauma was assessed with the Dutch version of the Childhood Trauma Questionnaire Short Form (CTQ) [53]. The short CTQ consists of 25 items rated on a 5-point Likert scale (1 = never true to 5 = very often true) inquiring about traumatic experiences in childhood. Five types of childhood maltreatment were assessed: emotional, physical and sexual abuse, and emotional and physical neglect, with five questions covering each type of trauma. As a general measure of childhood trauma a summary score was created by adding the mean scores of each trauma type. The CTQ data were missing for two persons (0.7% missing data), as described in a previous study [29].

Level of urbanicity

We generated a historical population density record for each municipality from 1930 onwards using the Dutch Central Bureau of Statistics (CBS) and equivalent Belgium database. When data was not available, linear extrapolations were computed. When historical names of municipalities disappeared from historical records (e.g. due to city mergers) we used the available data from the agglomerate city. Subjects were asked to describe where they lived during their life. This resulted in a number of records for each subject, containing locations by age period. For each of these records, we computed the average population density (by square kilometer, excluding water) of the municipality for the matching year periods. Subsequently, it was determined where the subject lived at birth, between ages 0–4 years; between 5–9 years; 10–14 years; 15–19 years; 20–39 years; 40–59 years; and 60+ up to the actual age. Average population density over the period was categorized conform the Dutch CBS urbanicity rating (1 = $<500/km^2$; 2 = $500–1000/km^2$; 3 = $1000–1500/km^2$; 4 = $1500–2500/km^2$; 5 = $2500+/km^2$). The periods 0–4 years, 5–9 years and 10–14 years were merged to average urbanicity exposure between 0–14 years. The latter divided by its tertiles was used as the primary variable reflecting developmental urbanicity exposure (low, medium, high) in the analyses. Urbanicity data were available for 263 participants (3.0% missing data). This urbanicity measures was also described in a previous paper [54].

MRI acquisition and processing

The MRI scans were acquired using a 3T Siemens scanner (Erlangen, Germany) and the following acquisition parameters: Modified Driven Equilibrium Fourier Transform (MDEFT)

sequence = 176 slices, 1 mm isotropic voxel size, echo time 2.4 ms, repetition time 7.92 ms, inversion time 910 ms, flip angle 15˚, total acquisition time 12 min 51 s; Magnetization Prepared Rapid Acquisition Gradient-Echo (MPRAGE; Alzheimer's Disease Neuroimaging Initiative) sequence = 192 slices, 1 mm isotropic voxel size, echo time 2.6 ms, repetition time 2250 ms, inversion time 900 ms, flip angle 9˚, total acquisition time 7 min 23 s. For both anatomical scans, the matrix size was 256 × 256 and field of view was 256 × 256 mm^2. The number of excitations was one. Two sequences were used because of a scanner update during data collection. The total proportion of MPRAGE scans did not differ between the groups ($\chi^2 = 4.95$, $p = 0.08$).

Cortical thickness measurement

Scans were processed and analyzed using Freesurfer stable release v5.3.0, http://surfer.nmr.mgh.harvard.edu [55–61]. To measure CT, the cerebral cortex was parcellated into units based on gyral and sulcal structure [62, 63]. Furthermore, a variety of surface-based data was created including maps of curvature and sulcal depth. This method used both intensity and continuity information from the entire three dimensional MR volume in segmentation and deformation procedures to produce representations of CT, calculated as the closest distance from the grey/white matter boundary to the grey matter/CSF boundary at each vertex on the tessellated surface [56]. The maps were created using spatial intensity gradients across tissue classes and were not restricted to the voxel resolution of the original data, thus were capable of detecting sub-millimeter differences between groups. CT measurement procedures have been validated against histological analysis [64] and manual measurements [18, 65]. Per hemisphere there are 34 regions (68 in total) of interest (adapted from the Desikant atlas) [62]; for each of these regions, CT measurements were derived by FreeSurfer and exported to Stata and R version 3.2.1. Thus, every individual had 68 CT measurements over the predefined ROIs. These procedures are described in detail in a previous paper [29].

Statistical/data analyses

The CT data consist of multiple observations nested within persons and within families, generating 3 levels of analysis: 68 regional CT measures (level 1) nested in subjects (level 2) who were part of the same families (level 3) [66]. Given the multilevel hierarchical structure of the data, compromising statistical independence of the observations, multilevel linear regression analyses were performed with CT as the dependent variable and subject number and family ID as random effects in all analyses. R version 3.2.1 [67] was used, employing the nlme, car and multcomp packages. Since the outcome (CT) represents means (based on varying numbers of vertices), the analyses were weighted based on the number of vertices per region. The regression coefficients (B) from the multilevel linear regression model represent the effect size of the predictors and can be interpreted identically to estimates in equivalent unilevel linear regression analyses. In order to check assumption of normality qq-plots of the residuals were inspected, which showed an even distribution. Also, the spread of standardized residuals against fitted values was evaluated, which was constant, i.e. there was no evidence of heteroscedasticity.

In order to study whether there is differential sensitivity to developmental urbanicity in the three groups and in both sexes, a three-way interaction between group (healthy control subject = 0, sibling = 1 or patient = 2, entered as linear variable and as dummy variables with healthy control subject as the reference category), sex (male = 0 or female = 1) and developmental urbanicity (low = 0, medium = 1 or high = 2, entered both as a linear variable and as dummy variables with 'low' urbanicity as the reference category) was added to the model. Main effects and interaction terms were tested using the chi-square test. The statistical model was:

$CT = \beta_0 + \beta_1(\text{group}) + \beta_2(\text{sex}) + \beta_3(\text{urbanicity}) + \beta_4(\text{group} \times \text{sex}) + \beta_5(\text{group} \times \text{urbanicity}) + \beta_6(\text{sex} \times \text{urbanicity}) + \beta_7(\text{group} \times \text{sex} \times \text{urbanicity})$. First, the three-way interaction was tested, and subsequently all the two-way interactions. Group was modelled both as linear and as dummy variables, with healthy control subjects as the reference category, in order to examine the level of dose-response. Developmental urbanicity was similarly entered both as a linear variable and as dummy variables, with 'low' urbanicity as the reference category, in order to examine the level of dose-response. In the case of significant interaction, individual slopes were calculated by combination of effects from the model and tested whether they differed significantly. All analyses were adjusted for the a priori selected confounders: age, sex, highest accomplished level of education and scantype (MDEFT or MPRAGE). The model was also analyzed with cannabis and childhood trauma as additional confounders, because cannabis and childhood trauma have been related to changes in CT in psychotic disorder [29] and to risk for psychotic disorder.

Results

Participant characteristics

All relevant data are provided in a supporting information file. Sociodemographic and clinical characteristics of the sample are summarized in Table 1. The patient group comprised more men and level of education was lowest in the patient group compared to controls and siblings. Seventy-four out of 89 patients were currently using AP medication (first generation AP: n = 3; second generation AP: n = 71), 16 patients used antidepressants, nine used benzodiazepines, six used antiepileptic medication and three used lithium. Two control subjects and three siblings used antidepressants, and one sibling used benzodiazepines. Cumulative lifetime AP exposure was not significantly associated with CT in the patient group ($B < 0.001$, $p = 0.55$).

Three-way interaction with group, sex and developmental urbanicity in the model of CT

The three-way interaction between group (linear variable and dummy variables), urbanicity (linear variable and dummy variables) and sex, in the model of CT was not significant (group linear variable × urbanicity linear variable × sex: $\chi^2 = 3.14$, $p = 0.08$; group linear variable × urbanicity dummy variables × sex: $\chi^2 = 2.88$, $p = 0.24$; group dummy variables × urbanicity linear variable × sex: $\chi^2 = 3.73$, $p = 0.16$; group dummy variables × urbanicity dummy variables x sex: $\chi^2 = 3.08$, $p = 0.55$), indicating that the association between CT and group was not moderated by developmental urbanicity and sex (see Table 2). Results did not change significantly when cannabis and childhood trauma were included in the analysis.

Two-way interaction between group and developmental urbanicity in the model of CT

There was no significant group (linear variable and dummy variables) × urbanicity (linear variable and dummy variables) interaction (group linear variable × urbanicity linear variable: $\chi^2 = 0.43$, $p = 0.51$; group linear variable × urbanicity dummy variables: $\chi^2 = 2.29$, $p = 0.32$; group dummy variables × urbanicity linear variable: $\chi^2 = 0.51$, $p = 0.77$; group dummy variables × urbanicity dummy variables: $\chi^2 = 4.23$, $p = 0.38$). Table 3 shows the mean values of CT per group and urbanicity level. There was no consistent pattern of reduction or increase in CT with increasing levels of developmental urbanicity in any group. Adding cannabis and childhood trauma as confounders did not change the results.

Table 1. Participant demographics.

	Patients (n = 89)	Sibling (n = 95)	Controls (n = 87)
Age at scan	28.1 ± 7.0	29.5 ± 8.8	30.8 ± 10.8
Sex n (%), male	60 (67%)	49 (52%)	33 (38%)
Level of education	4.3 ± 2.0	5.1 ± 2.0	5.4 ± 1.8
Cannabis lifetime[a]	45.0 ± 46.7	20.0 ± 37.7	7.4 ± 21.1
Other drug lifetime[a]	44.6 ± 88.7	6.1 ± 31.3	2.0 ± 11.6
Alcohol use units/week[b]	4.9 ± 9.0	9.6 ± 17.1	5.0 ± 7.0
Childhood urbanicity[c]	2.4 ± 1.3	2.3 ± 1.4	2.4 ± 1.5
Childhood trauma[d]	7.2 ± 2.8	5.8 ± 1.5	5.7 ± 1.8
PANSS Positive	10.5 ± 5.0	7.4 ± 1.5	7.3 ± 1.1
PANSS Negative	12.0 ± 5.7	8.5 ± 2.1	8.2 ± 1.0
PANSS Disorganization	12.5 ± 4.0	10.4 ± 0.9	10.2 ± 1.1
PANSS Excitement	9.8 ± 2.7	8.6 ± 1.4	8.3 ± 1.1
PANSS Emotional Distress	13.2 ± 5.2	10.0 ± 2.8	9.2 ± 2.0
Age of onset	21.7 ± 7.0	-	-
Antipsychotics[e]	6777.4 ± 6152.5	-	-

PANSS = Positive and negative syndrome scale.

[a] Mean number of times

[b] Weekly consumptions on the last 12 months.

[c] Mean level of childhood urban exposure, Five levels of urbanicity/population density 1 = <500 inhabitants/km^2; 2 = 500–1000 inhabitants/km^2; 3 = inhabitants 1000–1500/km^2; 4 = inhabitants 1500–2500/km^2; 5 = 2500+/km^2.

[d] Summary score of Childhood Trauma Questionnaire Short Form (CTQ)

[e] Lifetime exposure in haloperidol equivalents

doi:10.1371/journal.pone.0166651.t001

The other two-way interactions, group × sex and urbanicity × sex, were also not significant (group × sex [both dummy variables]: $\chi^2 = 0.73$, p = 0.69; urbanicity [linear variable] × sex (dummy variables): $\chi^2 = 0.35$, p = 0.55).

Main effects of group and urbanicity on CT

CT was significantly smaller in the patients compared to the controls and siblings (P vs. Con: B = -0.043, p<0.001; P vs. Sib: B = -0.031, p = 0.006). The siblings were not significantly different from the controls (B = -0.013, p = 0.31). There was neither a significant association

Table 2. Cortical Thickness as a Function of Group Status, Sex and Urbanicity.

	Urbanicity level	Patients		Relatives		Controls	
		No. obs (n)[a]	CT[b]	No. obs (n)	CT	No. obs (n)	CT
Male	Low	1360 (20)	2.50 ± 0.39	1224 (18)	2.47 ± 0.40	680 (10)	2.51 ± 0.42
	Medium	1564 (23)	2.50 ± 0.41	884 (13)	2.51 ± 0.38	816 (12)	2.56 ± 0.39
	High	1088 (16)	2.49 ± 0.41	1156 (17)	2.48 ± 0.38	612 (9)	2.49 ± 0.40
female	Low	340 (5)	2.47 ± 0.39	1156 (17)	2.53 ± 0.40	1564 (23)	2.50 ± 0.40
	Medium	680 (10)	2.52 ± 0.41	1156 (17)	2.54 ± 0.39	680 (10)	2.52 ± 0.39
	High	884 (13)	2.50 ± 0.41	680 (10)	2.55 ± 0.42	1360 (20)	2.48 ± 0.38

[a] No. obs = number of subjects × number of regions (68); numbers in parentheses indicate number of subjects.

[b] Mean ± SD, Means are raw means not adjusted for covariates.

doi:10.1371/journal.pone.0166651.t002

Table 3. Cortical Thickness as a Function of Group Status and Urbanicity.

	Patients		Relatives		Controls	
Urbanicity level	No. obs (n)[a]	CT[b]	No. obs (n)	CT	No. obs (n)	CT
Low	1700 (25)	2.50 ± 0.39	2380 (35)	2.50 ± 0.40	2244 (33)	2.51 ± 0.40
Medium	2244 (33)	2.51 ± 0.41	2040 (30)	2.53 ± 0.39	1496 (22)	2.54 ± 0.39
High	1972 (29)	2.50 ± 0.41	1836 (27)	2.50 ± 0.40	1972 (29)	2.49 ± 0.38

[a] No. obs = number of subjects × number of regions (68); numbers in parentheses indicate number of subjects.

[b] Mean ± SD, Means are raw means not adjusted for covariates.

doi:10.1371/journal.pone.0166651.t003

Table 4. Cortical Thickness by Group and Urbanicity level.

	No. of observations[a]	Cortical thickness Mean[b] ± SD	B[c]	p
**Group**				
Controls	5916 (87)	2.51 ± 0.40		
Siblings	6460 (95)	2.51 ± 0.40	-0.013	0.31
Patients	6052 (89)	2.50 ± 0.40	-0.043	<0.01
B linear trend[d]			-0.022	<0.01
**Urbanicity**				
Low	6324 (93)	2.50 ± 0.40		
Medium	5780 (85)	2.52 ± 0.40	0.001	0.96
High	5780 (85)	2.50 ± 0.40	0.001	0.91
B linear trend[d]			0.001	0.91

[a] No. of observations = number of subjects × number of regions (68); numbers in parentheses indicate number of subjects.

[b] Means are raw means not adjusted for covariates.

[c] B represent the regression coefficients from multilevel linear regression analyses, adjusted for age, sex, cannabis use and highest level of education, for group controls were the reference category and for urbanicity 'low' urbanicity level was the reference category

[d] B linear trend represents the summary change in CT with one unit increase in exposure level (group and urbanicity)

doi:10.1371/journal.pone.0166651.t004

between sex and CT (B = 0.004, p = 0.71), nor between developmental urbanicity (linear variable) and CT ($B < 0.001$, p = 0.91). See Table 4.

Post-hoc subgroup analysis

Subgroup analysis in which only 50% of the patients with the highest PANSS positive scores were included (n = 44), did not affect the pattern of the findings. The three-way interaction between urbanicity (linear variable and dummy variables), group and sex, and the two-way interaction between urbanicity (linear variable and dummy variables) and group were not significant (linear variable: three-way interaction: $\chi^2 = 4.97$, p = 0.08; two-way interaction: $\chi^2 = 1.38$, p = 0.50; dummy variables: three-way interaction: $\chi^2 = 5.20$, p = 0.27; two-way interaction: $\chi^2 = 5.51$, p = 0.24).

Discussion

The current study showed that urban upbringing was not associated with reductions of CT in individuals with (risk for) psychotic disorder or healthy controls.

To our knowledge no previous studies on the association between developmental urban exposure levels and CT as a function of different levels of psychosis risk are available. However, an fMRI study linked early life urbanicity exposure to differential activity of the pACC in social

stress processing in healthy students [32], suggesting that social stress mediates the impact of urbanicity on neural circuits, although other mechanisms can be envisaged. In the current study, there was no evidence for CT alterations in the pACC and the cingulate cortex. However, absence of structural alterations does not automatically imply absence of functional alterations: cell composition, cell activity or connectivity may be different despite normal volumes of discrete brain areas.

Epidemiological studies demonstrate that there is reason to believe that the relationship between urban upbringing and psychotic disorders is causal: (1) the findings are consistent across countries and cultures; (2) there is evidence of a dose-response relationship; and (3) urban upbringing precedes psychotic disorder [3]. The current study revealed that CT reductions in patients with psychotic disorder are not moderated by developmental urbanicity. An explanation for the absence of an association between cortical thinning and developmental urbanicity in psychotic disorder is that urban upbringing may influence different neurobiologic pathways that do not imply CT alteration. For example, changes in white matter could be involved or the nature of change could be functional instead of structural. In case of the latter, a potential pathologic effect of the proxy environmental risk factor associated with urbanicity on brain structure may be identified with functional MRI [32].

The absence of an association could also be explained by a small urban-rural difference. Our participants grew up in the Netherlands, which can be described as a relative safe and well developed country compared to certain other counties where the urban-rural discrepancies may be more prominent. However, studies conducted in the Netherlands did report a higher incidence of psychotic disorders related to urban upbringing [8, 68], indicating that although the difference is relatively small, it is still large enough to show an effect. Another explanation of the absence of a significant association could be that the number of participants in the higher urbanicity levels was relatively small compared to the lower urbanicity levels, resulting in reduced power.

Environmental factors for psychotic disorder, other than the urban exposure, have been related to grey matter reductions. For instance, a study by Cannon et al. [30] found that a history of fetal hypoxia is associated with gray matter volume reductions among patients with schizophrenia and their healthy siblings, but not among healthy controls. A plausible explanation for the relationship between this environmental exposure and gray matter reductions is that fetal hypoxia is more likely to have a direct effect on neurodevelopment than the developmental urban exposure since more than 50% of the candidate genes for susceptibility to schizophrenia are subject to regulation by hypoxia [69]. In addition, fetal hypoxia has been associated with increased brain dopamine synthesis capacity and concentrations [70]. In previous analyses on (partly) the same study sample as the current one, Habets and colleagues [29] found that higher levels of cannabis consumption (lifetime) and childhood trauma experiences were associated with increased cortical thinning in patients with psychotic disorder, but not in controls. In siblings, the same pattern was found for cannabis, but not for childhood trauma. Just as fetal hypoxia, cannabis exposure and developmental trauma have been more directly related to altered neurodevelopment than developmental urbanicity. Tetrahydrocannabinol (THC), the active psychotropic ingredient of cannabis, may lead to increased striatal dopamine levels [71]. THC is also believed to affect synaptic plasticity and it may affect gray matter volume through a mechanism on neurotoxicity [71, 72]. Childhood trauma is thought to affect dopaminergic circuits through altered functioning of the hypothalamic-pituitary-adrenal (HPA) axis [41, 42]. In conclusion, the developmental urbanicity exposure may be too crude compared to the other environmental risk factors to increase cerebral cortical vulnerability in individuals with (risk for) psychotic disorder.

Methodological considerations

This is the first study examining CT as the outcome of developmental urbanicity exposure and psychosis risk, in a relatively large study sample. Our study population is larger than most previous studies on CT in psychotic disorder [16, 18, 20, 22], although some studies had larger sample sizes e.g. the study by Goldman and colleagues [19]. Further, the absence of a main effect of urbanicity does not necessarily imply insufficient power for interaction: environmental risks may only become apparent when examined in a G × E model.

Population density was used as a measure of developmental urbanicity, but it is not clear whether this is a good measure of urban exposure. When the factors mediating the effect of the urbanicity exposure could be identified, the chance of finding underlying neuropathologic mechanisms may increase. Future research is needed to unravel the factors related to the urban environment that mediate the higher risk for psychotic disorders.

Systematic reviews and a longitudinal study [51, 73–75] suggest that AP treatment contributes to the brain structural changes observed in psychotic disorders. However, there was no evidence for an association between cumulative AP dose and CT in the current sample. It is thus unlikely that this has influenced our results.

Supporting Information

S1 Data. Supporting Data.
(XLS)

Acknowledgments

We wish to thank all participants and contributing staff of the participating mental health care centres. We thank Truda Driesen and Inge Crolla for their coordinating roles in the data collection, as well as the G.R.O.U.P. investigators: Richard Bruggeman, Wiepke Cahn, Lieuwe de Haan, René Kahn, Carin Meijer, Inez Myin-Germeys Jim van Os, Durk Wiersma.

Author Contributions

Conceptualization: AF JO PH EG MM RL.

Data curation: AF PH EG MM.

Formal analysis: AF JO MM.

Funding acquisition: JO MM.

Investigation: PH EG MM.

Methodology: AF JO EG MM.

Project administration: MM.

Resources: AF JO PH EG MM.

Software: EG.

Supervision: JO MM.

Validation: AF JO EG MM.

Visualization: AF MM.

Writing – original draft: AF MM RL.

Writing – review & editing: AF MM RL.

References

1. Kelly BD, O'Callaghan E, Waddington JL, Feeney L, Browne S, Scully PJ, et al. Schizophrenia and the city: A review of literature and prospective study of psychosis and urbanicity in Ireland. Schizophrenia research. 2010; 116(1):75–89. Epub 2009/11/10. doi: 10.1016/j.schres.2009.10.015 PMID: 19897342
2. McGrath J, Saha S, Welham J, El Saadi O, MacCauley C, Chant D. A systematic review of the incidence of schizophrenia: the distribution of rates and the influence of sex, urbanicity, migrant status and methodology. BMC Med. 2004; 2:13. Epub 2004/04/30. doi: 10.1186/1741-7015-2-13 PMID: 15115547
3. Krabbendam L, van Os J. Schizophrenia and urbanicity: a major environmental influence—conditional on genetic risk. Schizophrenia bulletin. 2005; 31(4):795–9. Epub 2005/09/10. doi: 10.1093/schbul/sbi060 PMID: 16150958
4. McGrath J, Scott J. Urban birth and risk of schizophrenia: a worrying example of epidemiology where the data are stronger than the hypotheses. Epidemiologia e psichiatria sociale. 2006; 15(4):243–6. Epub 2007/01/06. PMID: 17203614
5. Pedersen CB, Mortensen PB. Evidence of a dose-response relationship between urbanicity during upbringing and schizophrenia risk. Archives of general psychiatry. 2001; 58(11):1039–46. Epub 2001/12/26. PMID: 11695950
6. March D, Hatch SL, Morgan C, Kirkbride JB, Bresnahan M, Fearon P, et al. Psychosis and place. Epidemiol Rev. 2008; 30:84–100. Epub 2008/08/02. doi: 10.1093/epirev/mxn006 PMID: 18669521
7. Vassos E, Pedersen C, Murray R, Collier D, Lewis C. A meta-analysis of the association of urbanicity with schizophrenia. Schiz Bull. 2012; in press.
8. Marcelis M, Takei N, van Os J. Urbanization and risk for schizophrenia: does the effect operate before or around the time of illness onset? Psychological medicine. 1999; 29(5):1197–203. Epub 1999/11/27. PMID: 10576311
9. Pedersen CB, Mortensen PB. Why factors rooted in the family may solely explain the urban-rural differences in schizophrenia risk estimates. Epidemiologia e psichiatria sociale. 2006; 15(4):247–51. PMID: 17203615
10. Van Os J, Hanssen M, Bak M, Bijl RV, Vollebergh W. Do urbanicity and familial liability coparticipate in causing psychosis? Am J Psychiatry. 2003; 160(3):477–82. doi: 10.1176/appi.ajp.160.3.477 PMID: 12611828
11. van Os J, Pedersen CB, Mortensen PB. Confirmation of synergy between urbanicity and familial liability in the causation of psychosis. Am J Psychiatry. 2004; 161(12):2312–4. doi: 10.1176/appi.ajp.161.12.2312 PMID: 15569906
12. Weiser M, van Os J, Reichenberg A, Rabinowitz J, Nahon D, Kravitz E, et al. Social and cognitive functioning, urbanicity and risk for schizophrenia. The British journal of psychiatry: the journal of mental science. 2007; 191:320–4. Epub 2007/10/02.
13. van Os J, Driessen G, Gunther N, Delespaul P. Neighbourhood variation in incidence of schizophrenia. Evidence for person-environment interaction. The British journal of psychiatry: the journal of mental science. 2000; 176:243–8. Epub 2001/02/07.
14. Selten JP, Cantor-Graae E. Hypothesis: social defeat is a risk factor for schizophrenia? The British journal of psychiatry Supplement. 2007; 51:s9–12. Epub 2008/01/19. doi: 10.1192/bjp.191.51.s9 PMID: 18055945
15. Byun MS, Kim JS, Jung WH, Jang JH, Choi JS, Kim SN, et al. Regional cortical thinning in subjects with high genetic loading for schizophrenia. Schizophrenia research. 2012; 141(2–3):197–203. Epub 2012/09/25. doi: 10.1016/j.schres.2012.08.028 PMID: 22998933
16. Jung WH, Kim JS, Jang JH, Choi JS, Jung MH, Park JY, et al. Cortical thickness reduction in individuals at ultra-high-risk for psychosis. Schizophrenia bulletin. 2011; 37(4):839–49. Epub 2009/12/23. doi: 10.1093/schbul/sbp151 PMID: 20026559
17. Goghari VM, Rehm K, Carter CS, MacDonald AW 3rd. Regionally specific cortical thinning and gray matter abnormalities in the healthy relatives of schizophrenia patients. Cerebral cortex. 2007; 17(2):415–24. Epub 2006/03/21. doi: 10.1093/cercor/bhj158 PMID: 16547347
18. Kuperberg GR, Broome MR, McGuire PK, David AS, Eddy M, Ozawa F, et al. Regionally localized thinning of the cerebral cortex in schizophrenia. Archives of general psychiatry. 2003; 60(9):878–88. Epub 2003/09/10. doi: 10.1001/archpsyc.60.9.878 PMID: 12963669
19. Goldman AL, Pezawas L, Mattay VS, Fischl B, Verchinski BA, Chen Q, et al. Widespread reductions of cortical thickness in schizophrenia and spectrum disorders and evidence of heritability. Archives of

general psychiatry. 2009; 66(5):467–77. Epub 2009/05/06. doi: 10.1001/archgenpsychiatry.2009.24 PMID: 19414706

20. Cahn W, Hulshoff Pol HE, Lems EB, van Haren NE, Schnack HG, van der Linden JA, et al. Brain volume changes in first-episode schizophrenia: a 1-year follow-up study. Archives of general psychiatry. 2002; 59(11):1002–10. PMID: 12418933

21. Fornito A, Yucel M, Patti J, Wood SJ, Pantelis C. Mapping grey matter reductions in schizophrenia: an anatomical likelihood estimation analysis of voxel-based morphometry studies. Schizophrenia research. 2009; 108(1–3):104–13. doi: 10.1016/j.schres.2008.12.011 PMID: 19157788

22. Gutierrez-Galve L, Wheeler-Kingshott CA, Altmann DR, Price G, Chu EM, Leeson VC, et al. Changes in the frontotemporal cortex and cognitive correlates in first-episode psychosis. Biological psychiatry. 2010; 68(1):51–60. Epub 2010/05/11. doi: 10.1016/j.biopsych.2010.03.019 PMID: 20452574

23. Glahn DC, Laird AR, Ellison-Wright I, Thelen SM, Robinson JL, Lancaster JL, et al. Meta-analysis of gray matter anomalies in schizophrenia: application of anatomic likelihood estimation and network analysis. Biological psychiatry. 2008; 64(9):774–81. doi: 10.1016/j.biopsych.2008.03.031 PMID: 18486104

24. Oertel-Knochel V, Knochel C, Rotarska-Jagiela A, Reinke B, Prvulovic D, Haenschel C, et al. Association between psychotic symptoms and cortical thickness reduction across the schizophrenia spectrum. Cerebral cortex. 2013; 23(1):61–70. Epub 2012/02/01. doi: 10.1093/cercor/bhr380 PMID: 22291030

25. Honea R, Crow TJ, Passingham D, Mackay CE. Regional deficits in brain volume in schizophrenia: a meta-analysis of voxel-based morphometry studies. The American journal of psychiatry. 2005; 162 (12):2233–45. doi: 10.1176/appi.ajp.162.12.2233 PMID: 16330585

26. Boos HB, Cahn W, van Haren NE, Derks EM, Brouwer RM, Schnack HG, et al. Focal and global brain measurements in siblings of patients with schizophrenia. Schizophrenia bulletin. 2012; 38(4):814–25. doi: 10.1093/schbul/sbq147 PMID: 21242319

27. Honea RA, Meyer-Lindenberg A, Hobbs KB, Pezawas L, Mattay VS, Egan MF, et al. Is gray matter volume an intermediate phenotype for schizophrenia? A voxel-based morphometry study of patients with schizophrenia and their healthy siblings. Biological psychiatry. 2008; 63(5):465–74. doi: 10.1016/j.biopsych.2007.05.027 PMID: 17689500

28. Goldman AL, Pezawas L, Mattay VS, Fischl B, Verchinski BA, Zoltick B, et al. Heritability of brain morphology related to schizophrenia: a large-scale automated magnetic resonance imaging segmentation study. Biological psychiatry. 2008; 63(5):475–83. doi: 10.1016/j.biopsych.2007.06.006 PMID: 17727823

29. Habets P, Marcelis M, Gronenschild E, Drukker M, van Os J. Reduced cortical thickness as an outcome of differential sensitivity to environmental risks in schizophrenia. Biological psychiatry. 2011; 69(5):487–94. Epub 2010/10/19. doi: 10.1016/j.biopsych.2010.08.010 PMID: 20951979

30. Cannon TD, van Erp TG, Rosso IM, Huttunen M, Lonnqvist J, Pirkola T, et al. Fetal hypoxia and structural brain abnormalities in schizophrenic patients, their siblings, and controls. Archives of general psychiatry. 2002; 59(1):35–41. PMID: 11779280

31. Haddad L, Schafer A, Streit F, Lederbogen F, Grimm O, Wust S, et al. Brain Structure Correlates of Urban Upbringing, an Environmental Risk Factor for Schizophrenia. Schizophrenia bulletin. 2014.

32. Lederbogen F, Kirsch P, Haddad L, Streit F, Tost H, Schuch P, et al. City living and urban upbringing affect neural social stress processing in humans. Nature. 2011; 474(7352):498–501. Epub 2011/06/24. doi: 10.1038/nature10190 PMID: 21697947

33. Tomoda A, Suzuki H, Rabi K, Sheu YS, Polcari A, Teicher MH. Reduced prefrontal cortical gray matter volume in young adults exposed to harsh corporal punishment. NeuroImage. 2009; 47 Suppl 2:T66–71.

34. Tomoda A, Polcari A, Anderson CM, Teicher MH. Reduced visual cortex gray matter volume and thickness in young adults who witnessed domestic violence during childhood. PloS one. 2012; 7(12): e52528. doi: 10.1371/journal.pone.0052528 PMID: 23300699

35. Frodl T, Reinhold E, Koutsouleris N, Reiser M, Meisenzahl EM. Interaction of childhood stress with hippocampus and prefrontal cortex volume reduction in major depression. Journal of psychiatric research. 2010; 44(13):799–807. doi: 10.1016/j.jpsychires.2010.01.006 PMID: 20122698

36. Schubert MI, Porkess MV, Dashdorj N, Fone KC, Auer DP. Effects of social isolation rearing on the limbic brain: a combined behavioral and magnetic resonance imaging volumetry study in rats. Neuroscience. 2009; 159(1):21–30. doi: 10.1016/j.neuroscience.2008.12.019 PMID: 19141315

37. Jackowski A, Perera TD, Abdallah CG, Garrido G, Tang CY, Martinez J, et al. Early-life stress, corpus callosum development, hippocampal volumetrics, and anxious behavior in male nonhuman primates. Psychiatry research. 2011; 192(1):37–44. doi: 10.1016/j.pscychresns.2010.11.006 PMID: 21377844

38. Duque A, Coman D, Carlyle BC, Bordner KA, George ED, Papademetris X, et al. Neuroanatomical changes in a mouse model of early life neglect. Brain structure & function. 2012; 217(2):459–72.

39. Spinelli S, Chefer S, Suomi SJ, Higley JD, Barr CS, Stein E. Early-life stress induces long-term morphologic changes in primate brain. Archives of general psychiatry. 2009; 66(6):658–65. doi: 10.1001/archgenpsychiatry.2009.52 PMID: 19487631

40. Lu S, Gao W, Wei Z, Wu W, Liao M, Ding Y, et al. Reduced cingulate gyrus volume associated with enhanced cortisol awakening response in young healthy adults reporting childhood trauma. PloS one. 2013; 8(7):e69350. doi: 10.1371/journal.pone.0069350 PMID: 23894454

41. Heim C, Newport DJ, Heit S, Graham YP, Wilcox M, Bonsall R, et al. Pituitary-adrenal and autonomic responses to stress in women after sexual and physical abuse in childhood. JAMA: the journal of the American Medical Association. 2000; 284(5):592–7.

42. Walker EF, Diforio D. Schizophrenia: a neural diathesis-stress model. Psychological review. 1997; 104 (4):667–85. PMID: 9337628

43. Luo Y, Umegaki H, Wang X, Abe R, Roth GS. Dopamine induces apoptosis through an oxidation-involved SAPK/JNK activation pathway. The Journal of biological chemistry. 1998; 273(6):3756–64. PMID: 9452508

44. Luo Y, Hattori A, Munoz J, Qin ZH, Roth GS. Intrastriatal dopamine injection induces apoptosis through oxidation-involved activation of transcription factors AP-1 and NF-kappaB in rats. Molecular pharmacology. 1999; 56(2):254–64. PMID: 10419543

45. Wersinger C, Chen J, Sidhu A. Bimodal induction of dopamine-mediated striatal neurotoxicity is mediated through both activation of D1 dopamine receptors and autoxidation. Molecular and cellular neurosciences. 2004; 25(1):124–37. doi: 10.1016/j.mcn.2003.10.002 PMID: 14962746

46. Bora E, Fornito A, Yucel M, Pantelis C. The effects of gender on grey matter abnormalities in major psychoses: a comparative voxelwise meta-analysis of schizophrenia and bipolar disorder. Psychological medicine. 2011:1–13.

47. Maxwell ME. Genetics Initiative: Family Interview for Genetic Studies (FIGS): National Institute of Mental Health; 1992.

48. APA. Diagnostic and Statistical Manual of Mental Disorders. 4 ed. Washington, DC: American Psychiatric Association; 2000.

49. Andreasen NC, Flaum M, Arndt S. The Comprehensive Assessment of Symptoms and History (CASH). An instrument for assessing diagnosis and psychopathology. Archives of general psychiatry. 1992; 49 (8):615–23. Epub 1992/08/01. PMID: 1637251

50. Kay SR, Fiszbein A, Opler LA. The positive and negative syndrome scale (PANSS) for schizophrenia. Schizophrenia bulletin. 1987; 13(2):261–76. Epub 1987/01/01. PMID: 3616518

51. Navari S, Dazzan P. Do antipsychotic drugs affect brain structure? A systematic and critical review of MRI findings. Psychological medicine. 2009; 39(11):1763–77. doi: 10.1017/S0033291709005315 PMID: 19338710

52. WHO. Composite International Diagnostic Interview. Geneva: World Health Organization. 1990.

53. Bernstein DP, Ahluvalia T, Pogge D, Handelsman L. Validity of the Childhood Trauma Questionnaire in an adolescent psychiatric population. Journal of the American Academy of Child and Adolescent Psychiatry. 1997; 36(3):340–8. doi: 10.1097/00004583-199703000-00012 PMID: 9055514

54. Frissen A, Lieverse R, Drukker M, Delespaul P, Lataster T, Myin-Germeys I, et al. Evidence that childhood urban environment is associated with blunted stress reactivity across groups of patients with psychosis, relatives of patients and controls. Social psychiatry and psychiatric epidemiology. 2014; 49 (10):1579–87. doi: 10.1007/s00127-014-0859-3 PMID: 24643299

55. Dale AM, Fischl B, Sereno MI. Cortical surface-based analysis. I. Segmentation and surface reconstruction. NeuroImage. 1999; 9(2):179–94. doi: 10.1006/nimg.1998.0395 PMID: 9931268

56. Fischl B, Dale AM. Measuring the thickness of the human cerebral cortex from magnetic resonance images. Proceedings of the National Academy of Sciences of the United States of America. 2000; 97 (20):11050–5. doi: 10.1073/pnas.200033797 PMID: 10984517

57. Fischl B, Salat DH, Busa E, Albert M, Dieterich M, Haselgrove C, et al. Whole brain segmentation: automated labeling of neuroanatomical structures in the human brain. Neuron. 2002; 33(3):341–55. PMID: 11832223

58. Fischl B, Sereno MI, Dale AM. Cortical surface-based analysis. II: Inflation, flattening, and a surface-based coordinate system. NeuroImage. 1999; 9(2):195–207. doi: 10.1006/nimg.1998.0396 PMID: 9931269

59. Han X, Jovicich J, Salat D, van der Kouwe A, Quinn B, Czanner S, et al. Reliability of MRI-derived measurements of human cerebral cortical thickness: the effects of field strength, scanner upgrade and manufacturer. NeuroImage. 2006; 32(1):180–94. doi: 10.1016/j.neuroimage.2006.02.051 PMID: 16651008

60. Jovicich J, Czanner S, Greve D, Haley E, van der Kouwe A, Gollub R, et al. Reliability in multi-site structural MRI studies: effects of gradient non-linearity correction on phantom and human data. NeuroImage. 2006; 30(2):436–43. doi: 10.1016/j.neuroimage.2005.09.046 PMID: 16300968

61. Segonne F, Dale AM, Busa E, Glessner M, Salat D, Hahn HK, et al. A hybrid approach to the skull stripping problem in MRI. NeuroImage. 2004; 22(3):1060–75. doi: 10.1016/j.neuroimage.2004.03.032 PMID: 15219578

62. Desikan RS, Segonne F, Fischl B, Quinn BT, Dickerson BC, Blacker D, et al. An automated labeling system for subdividing the human cerebral cortex on MRI scans into gyral based regions of interest. NeuroImage. 2006; 31(3):968–80. doi: 10.1016/j.neuroimage.2006.01.021 PMID: 16530430

63. Fischl B, van der Kouwe A, Destrieux C, Halgren E, Segonne F, Salat DH, et al. Automatically parcellating the human cerebral cortex. Cerebral cortex. 2004; 14(1):11–22. PMID: 14654453

64. Rosas HD, Liu AK, Hersch S, Glessner M, Ferrante RJ, Salat DH, et al. Regional and progressive thinning of the cortical ribbon in Huntington's disease. Neurology. 2002; 58(5):695–701. PMID: 11889230

65. Salat DH, Buckner RL, Snyder AZ, Greve DN, Desikan RS, Busa E, et al. Thinning of the cerebral cortex in aging. Cerebral cortex. 2004; 14(7):721–30. doi: 10.1093/cercor/bhh032 PMID: 15054051

66. Schwartz JE, Stone AA. Strategies for analyzing ecological momentary assessment data. Health psychology: official journal of the Division of Health Psychology, American Psychological Association. 1998; 17(1):6–16. Epub 1998/02/12.

67. The R Foundation. https://www.r-project.org/.

68. Marcelis M, Navarro-Mateu F, Murray R, Selten JP, Van Os J. Urbanization and psychosis: a study of 1942–1978 birth cohorts in The Netherlands. Psychological medicine. 1998; 28(4):871–9. Epub 1998/09/02. PMID: 9723142

69. Schmidt-Kastner R, van Os J, WMS H, Schmitz C. Gene regulation by hypoxia and the neurodevelopmental origin of schizophrenia. Schizophrenia research. 2006; 84(2–3):253–71. doi: 10.1016/j.schres.2006.02.022 PMID: 16632332

70. Howes OD, Murray RM. Schizophrenia: an integrated sociodevelopmental-cognitive model. Lancet. 2014; 383(9929):1677–87. doi: 10.1016/S0140-6736(13)62036-X PMID: 24315522

71. Kuepper R, Morrison PD, van Os J, Murray RM, Kenis G, Henquet C. Does dopamine mediate the psychosis-inducing effects of cannabis? A review and integration of findings across disciplines. Schizophrenia research. 2010; 121(1–3):107–17. doi: 10.1016/j.schres.2010.05.031 PMID: 20580531

72. Stone JM, Day F, Tsagaraki H, Valli I, McLean MA, Lythgoe DJ, et al. Glutamate dysfunction in people with prodromal symptoms of psychosis: relationship to gray matter volume. Biological psychiatry. 2009; 66(6):533–9. doi: 10.1016/j.biopsych.2009.05.006 PMID: 19559402

73. Moncrieff J, Leo J. A systematic review of the effects of antipsychotic drugs on brain volume. Psychological medicine. 2010; 40(9):1409–22. doi: 10.1017/S0033291709992297 PMID: 20085668

74. Andreasen NC, Liu D, Ziebell S, Vora A, Ho BC. Relapse duration, treatment intensity, and brain tissue loss in schizophrenia: a prospective longitudinal MRI study. The American journal of psychiatry. 2013; 170(6):609–15. doi: 10.1176/appi.ajp.2013.12050674 PMID: 23558429

75. White T, Andreasen NC, Nopoulos P, Magnotta V. Gyrification abnormalities in childhood- and adolescent-onset schizophrenia. Biological psychiatry. 2003; 54(4):418–26. Epub 2003/08/14. PMID: 12915286
